# Supplementary material for: Connecting real-world digital mobility assessment to clinical outcomes for regulatory and clinical endorsement–the Mobilise-D study protocol
Source: PLoS One. 2022 Oct 6;17(10):e0269615. doi: 10.1371/journal.pone.0269615 (PMC9536536; doi:10.1371/journal.pone.0269615)
Supplement: S1 File — (PDF) [file pone.0269615.s002.pdf]

**This protocol has regard for the HRA guidance and order of content**

## FULL/LONG TITLE OF THE STUDY

Validating digital mobility assessment using wearable technology – the Mobilise-D Clinical Validation study.

## SHORT STUDY TITLE / ACRONYM

Mobilise-D – Clinical Validation Study

## PROTOCOL VERSION NUMBER AND DATE

Version 1.5 25 May 2021

## RESEARCH REFERENCE NUMBERS

| | |
|---|---|
| **IRAS Number:** | 289543 |
| **ISRCTN Number:** | 12051706 |
| **SPONSORS Number:** | 09672 |
| **FUNDERS Number:** | 820820 |

## SIGNATURE PAGE

The undersigned confirm that the following protocol has been agreed and accepted and that the Chief Investigator agrees to conduct the study in compliance with the approved protocol and will adhere to the principles outlined in the Declaration of Helsinki, Good Clinical Practise (GCP) guidelines, the Sponsor's (and any other relevant) SOPs, and other regulatory requirements.

I agree to ensure that the confidential information contained in this document will not be used for any other purpose other than the evaluation or conduct of the clinical investigation without the prior written consent of the Sponsor

I also confirm that I will make the findings of the study publically available through publication or other dissemination tools without any unnecessary delay and that an honest accurate and transparent account of the study will be given; and that any discrepancies and serious breaches of GCP from the study as planned in this protocol will be explained.

**For and on behalf of the Study Sponsor:**

Signature: .......................................................................................... Date: ....../....../......

Name (please print):

..........................................................................................

Position:
..........................................................................................

**Chief Investigator:**

Signature:
.......................................................................................... Date: ....../....../......

Name: (please print):

.........................................................................................

**(Optional)**

**Statistician:**

Signature:
..........................................................................................

Name: (please print):

..........................................................................................

Position:
..........................................................................................

## KEY STUDY CONTACTS

| | |
|---|---|
| Chief Investigator | Professor Lynn Rochester<br>Newcastle University<br>0191 208 1291<br>lynn.rochester@newcastle.ac.uk |
| Study Co-ordinator/s | Prof. Dr. Clemens Becker<br>Work Package Lead of Clinical Validation Study<br>Robert Bosch Gesellschaft fuer Medizinische Forschung<br>clemens.becker@rbk.de<br><br>Isabel Neatrour<br>Newcastle University<br>0191 208 1387<br>isabel.neatrour@newcastle.ac.uk<br><br>Dr. Carl-Philipp Jansen<br>Robert Bosch Gesellschaft fuer Medizinische Forschung<br>carl-philipp.jansen@rbk.de |
| Sponsor | Chris Price<br>The Newcastle upon Tyne Hospitals NHS Foundation Trust<br>0191 282 4461<br>Tnu-tr.sponsormanagement@nhs.net |
| Funder | European Commission via the Innovative Medicines Initiative (IMI) 2 Joint Undertaking.<br>This Joint Undertaking receives support from the European Union’s Horizon 2020 research and innovation programme and EFPIA.<br>0032 2 221 81 81<br>Infodesk@imi.europa.eu |
| Key Protocol Contributors: | **PD Cohort:**<br><br>Prof. Walter Maetzler (w.maetzler@neurologie.uni-kiel.de) Christian-Albrechts-Universität zu Kiel |

| | |
|---|---|
| | Prof. Daniela Berg (d.berg@neurologie.uni-kiel.de)<br>Christian-Albrechts-Universität zu Kiel<br>Cohort leads PD cohort<br><br>Dr. Christian Schlenstedt (c.schlenstedt@neurologie.uni-kiel.de)<br>Christian-Albrechts-Universität zu Kiel<br><br>Dr. Janet van Uem (janet.vanuem@uksh.de)<br>Christian-Albrechts-Universität zu Kiel<br><br>Prof. Alice Nieuwboer (alice.nieuwboer@kuleuven.be)<br>Katholieke Universiteit Leuven<br><br>Prof. Lynn Rochester (lynn.rochester@ncl.ac.uk**)**<br>University of Newcastle upon Tyne<br><br>Dr. Alison Yarnall (alison.yarnall@ncl.ac.uk)<br>University of Newcastle upon Tyne<br><br>Prof. Anat Mirelman (anatmi@tlvmc.gov.il)<br>Medical Research Foundation and Infrastructure Development Health Services<br><br>Prof. Jeff Hausdorff (jhausdor@tlvmc.gov.il**)**<br>Medical Research Foundation and Infrastructure Development Health Services<br><br>Prof. Jochen Klucken (jochen.klucken@uk-erlangen.de)<br>Universitätsklinikum Erlangen<br><br>Mark Gordon, MD (Mark.gordon@tevapharm.com)<br>Teva Pharmaceuticals |
| | **MS Cohort:**<br><br>Prof. Basil Sharrack (b.sharrack@sheffield.ac.uk)<br>The University of Sheffield<br>Cohort lead MS cohort<br><br>Prof. Letizia Leocani (leocani.letizia@hsr.it)<br>Università Vita-Salute San Raffaele<br><br>Prof. Giancarlo Comi (comi.giancarlo@hsr.it)<br>Università Vita-Salute San Raffaele<br><br>Dr Yann Hyvert (yann.hyvert@merckgroup.com)<br>Merck Kommanditgesellschaft auf Aktien |

| | |
|---|---|
| | **COPD Cohort:**<br><br>Prof. Thierry Troosters (thierry.troosters@kuleuven.be)<br>Katholieke Universiteit Leuven<br>Cohort lead COPD<br><br>Prof. Heleen Demeyer (heleen.demeyer@kuleuven.be)<br>Katholieke Universiteit Leuven<br>Cohort lead COPD<br><br>Prof. Judith Garcia-Aymerich (judith.garcia@isglobal.org)<br>Institut de Salut Global (ISGlobal) Barcelona<br><br>Dr. Elena Gimeno-Santos (elena.gimeno@isglobal.org)<br>Institut de Salut Global (ISGlobal) Barcelona<br><br>Prof. Ioannis Vogiatzis<br>(ioannis.vogiatzis@northumbria.ac.uk)<br>University of Northumbria at Newcastle<br><br>Dr. Nikolaos Chynkiamis<br>(n.chynkiamis@northumbria.ac.uk &<br>nikolaos.chynkiamis@gmail.com)<br>University of Northumbria at Newcastle & Thorax Research Foundation<br><br>Dr. Henrik Watz (h.watz@pulmoresearch.de)<br>Pneumologisches Forschungsinstitut an der LungenClinic Grosshansdorf GmbH<br><br>Prof. Milo Puhan (miloalan.puhan@uzh.ch)<br>Universität Zürich<br><br>Dr. Anja.Frei (anja.frei@uzh.ch)<br>Universität Zürich<br><br>Dr. Nicholas S Hopkinson (n.hopkinson@imperial.ac.uk)<br>Imperial College London<br><br>Professor Michael Polkey (m.polkey@rbht.nhs.uk)<br>Imperial College London |
| | **PFF Cohort:**<br><br>Prof. Clemens Becker (clemens.becker@rbk.de)<br>Robert Bosch Gesellschaft fuer Medizinische Forschung<br>PFF cohort lead |

| | |
|---|---|
| | Prof. Bernd Kinner (bernd.kinner@rbk.de)<br>Robert Bosch Gesellschaft fuer Medizinische Forschung<br><br>Dr. Kristin Taraldsen (kristin.taraldsen@ntnu.no)<br>Norges teknisk-naturvitenskaplige universitet<br><br>Prof. Lars-Gunnar.Johnsen (lars.gunnar.johnsen@ntnu.no)<br>Norges teknisk-naturvitenskaplige universitet<br><br>Prof. Ingvild Saltvedt (ingvild.saltvedt@ntnu.no)<br>Norges teknisk-naturvitenskaplige universitet<br><br>Prof. Jorunn Helbostad (jorunn.helbostad@ntnu.no)<br>Norges teknisk-naturvitenskaplige universitet<br><br>Prof. Hubert Blain (h-blain@chu-montpellier.fr)<br>Pôle de Gérontologie du CHU de Montpellier<br><br>Dr. Ram Miller (ram.miller@novartis.com)<br>Novartis Institutes for BioMedical Research |
| | **General:**<br>Dr. Stefanie Mikolaizak (s.mikolaizak@neura.edu.au)<br>Robert Bosch Gesellschaft für Medizinische Forschung<br>Manual Development and Training<br><br>Dr Lars Schwickert (lars.schwickert@rbk.de)<br>Robert Bosch Gesellschaft für Medizinische Forschung<br>Manual Development and Training<br><br>Prof. Beatrix Vereijken (beatrix.vereijken@ntnu.no)<br>Norges teknisk-naturvitenskaplige universitet<br>PPIE coordination and dissemination<br><br>Dr. Paolo Piraino (paolo.piraino@bayer.com)<br>Bayer AG |
| Data Management Lead | Prof. Brian Caulfield (b.caulfield@ucd.ie)<br>University College Dublin<br><br>Dr. David Singleton (d.singleton@ucd.ie)<br>University College Dublin<br><br>Dr. Heiko Gaßner (heiko.gassner@uk.erlangen.de)<br>Universitätsklinikum Erlangen |
| Statistician | Aida Aydemir (aida.aydemir@emdserono.com)<br>Merck Kommanditgesellschaft auf Aktien |
| Epidemiology | Prof. Judith Garcia-Aymerich (judith.garcia@isglobal.org) |

| | |
|---|---|
| | Institut De Salut Global (ISGlobal) Barcelona<br><br>Prof. Jochen Klenk (Jochen.Klenk@rbk.de)<br>Robert Bosch Gesellschaft fuer Medizinische Forschung |
| Committees | Study Management Group<br>Study Steering Committee |

## i. LIST of CONTENTS


| GENERAL INFORMATION | Page No. |
|---|---|
| TITLE PAGE | |
| RESEARCH REFERENCE NUMBERS | |
| SIGNATURE PAGE | |
| KEY STUDY CONTACTS | |
| i. LIST of CONTENTS | |
| ii. LIST OF ABBREVIATIONS | |
| iii. STUDY SUMMARY | |
| iv. FUNDING | |
| v. ROLE OF SPONSOR AND FUNDER | |
| vi. ROLES & RESPONSIBILITIES OF STUDY MANAGEMENT COMMITTEES, GROUPS AND INDIVIDUALS | |
| vii, PROTOCOL CONTRIBUTERS | |
| viii. KEYWORDS | |
| ix. STUDY FLOW CHART | |
| **SECTION** | |
| 1. BACKGROUND | |
| 2. RATIONALE | |
| 3. OBJECTIVES AND OUTCOME MEASURES/ENDPOINTS | |
| 4. STUDY DESIGN | |
| 5. STUDY SETTING | |
| 6. PARTICIPANT ELIGIBILITY CRITERIA | |
| 7. STUDY PROCEDURES | |
| 8. PHARMACOVIGILANCE | |
| 9. STATISTICS AND DATA ANALYSIS | |
| 10. DATA MANAGEMENT | |
| 11. MONITORING, AUDIT & INSPECTION | |
| 12. ETHICAL AND REGULATORY CONSIDERATIONS | |
| 13. DISSEMINATION POLICY | |
| 14. REFERENCES | |
| 15. APPENDICIES | |

## ii. LIST OF ABBREVIATIONS

| | |
|---|---|
| **BIA** | Bioelectrical Impedance Analysis |
| **CAT** | COPD Assessment Test |
| **CDR** | Clinical Dementia Rating |
| **COPD** | Chronic Obstructive Pulmonary Disease |
| **C-PPAC** | PROactive Physical Activity in COPD (clinical visit) |
| **DEMMI** | De Morton Mobility Index |
| **DMOs** | Digital Mobility Outcomes |
| **DMA** | Digital Mobility Assessment |
| **EDSS** | Expanded Disability Status Scale |
| **EFPIA** | European Federation of Pharmaceutical Industries and Associations |
| **EMA** | European Medicines Agency |
| **EQ-5D** | Euro-Qol |
| **FCI** | Functional Comorbidity Index |
| **FES-I** | Falls Efficacy Scale International |
| **FI** | Frailty Index |
| **FACIT** | Functional Assessment of Chronic Illness Therapy |
| **H&Y** | Hoehn & Yahr Staging of Parkinson's disease |
| **HADS** | Hospital Anxiety and Depression Scale |
| **LLFDI** | Late Life Functional Disability Index |
| **LSA** | Life Space Assessment |
| **MID** | Minimal Important Difference |
| **mMRC** | Modified Medical Research Council Dyspnoea Scale |
| **MMSE** | Mini-Mental State Examination |
| **MoCA** | Montreal Cognitive Assessment |
| **MS** | Multiple Sclerosis |
| **MSFC** | Multiple Sclerosis Functional Composite |
| **MSWS-12** | Multiple sclerosis walking scale-12 |
| **NEADL** | Nottingham Extended Activities of Daily Living |
| **NFOGQ** | New Freezing of Gait Questionnaire |
| **NHLSD** | Nursing Home Life Space Diameter |

| | |
|---|---|
| **NHS** | National Health Service |
| **PD** | Parkinson's disease |
| **PDDS** | Patient Determined Disease Steps |
| **PFF** | Proximal Femoral Fracture |
| **PHQ** | Patient Health Questionnaire |
| **PI** | Principal Investigator |
| **QTF** | Qualification Task Force |
| **QF** | Quadriceps Force |
| **REC** | Research Ethics Committee |
| **RWS** | Real-world walking speed |
| **SMG** | Study Management Group |
| **SPPB** | Short Physical Performance Battery |
| **SSC** | Study Steering Committee |
| **TUG** | Timed Up and Go |
| **UPDRS** | Unified Parkinson's Disease Rating Scale |
| **VAS** | Visual Analogue Scale |
| **4MWT** | 4 Metre Walking Test |
| **5CRT** | 5 chair rise test |
| **6MWT** | Six minute walking test |

## iii. STUDY SUMMARY

| | |
|---|---|
| Study Title | Validating digital mobility assessment using wearable technology – the Mobilise-D Clinical Validation study. |
| Internal ref. no. (or short title) | Mobilise-D - Clinical Validation Study |
| Study Design | Longitudinal Observational study |
| Study Participants | Participants will be recruited from four different disease cohorts; Patients diagnosed with Chronic Obstructive Pulmonary Disease (COPD), Parkinson's disease (PD) or Multiple Sclerosis (MS) or patients who have sustained a Proximal femoral fracture (PFF). |
| Planned Sample Size | 2,400 (600 from each cohort) |
| Follow up duration | 24 months |
| Planned Study Period | 31 months |

| | Objectives | Outcome Measures |
|---|---|---|
| Primary | Assess predictive capacity of DMOs | Global: LLFDI (functional)<br>PD: Fall frequency<br>MS: Fall frequency<br>COPD: Occurrence of moderate to severe COPD exacerbations<br>PFF: Admission to a care home |
| Secondary | Assess predictive capacity of DMOs | Global: hospital admission<br>PD: MDS-UPDRS III and II<br>MS: EDSS, T25-FW, MSWS-12<br>COPD: C-PPAC scores, time to first exacerbations, rate of exacerbations<br>PFF: SPPB, EQ-5D |
| | Assess construct validity of DMOs | Global: SPPB total, 6MWT<br>PD: MDS-UPDRS III, H&Y<br>MS: EDSS, T25-FW, MSWS-12, visual impairment<br>COPD: FEV1, mMRC dyspnea scale, CAT, C-PPAC, history of exacerbations<br>PFF: SPPB, MoCA, arterial hypertension and hearing loss |
| | Assess ability of DMOs to detect changes | Global: global anchor question<br>PD: MDS-UPDRS III<br>MS: EDSS<br>COPD: occurrence of exacerbations, mMRC, QF, C-PPAC<br>PFF: SPPB, LLFDI (disability component) |

| | | |
|---|---|---|
| | Estimate the MID of DMOs | Global: LLFDI total score<br>PD: MDS-UPDRS III and II<br>MS: T25-FW, MSWS-12<br>COPD: C-PPAC, CAT, 6MWD, steps/day<br>PFF: SPPB score, LLFDI (disability component) |
| | Describe real-world walking behaviour | Global: DMOs<br>PD: DMOs<br>MS: DMOs<br>COPD: DMOs<br>PFF: DMOs |

## iv. FUNDING AND SUPPORT IN KIND

This study is part of a larger project of work called Mobilise-D. Mobilise-D has received funding from the Innovative Medicines Initiative 2 Joint Understanding under grant agreement No 820820. This Joint Undertaking receives support from the European Union’s Horizon 2020 Research and Innovation Programme and European Federation of Pharmaceutical Industries and Associations.

## v. ROLE OF STUDY SPONSOR AND FUNDER

The Newcastle upon Tyne Hospitals NHS Foundation Trust (NuTH) will act as the Sponsor for the entire study, including sites based outside the UK in EU 27. As Sponsor, NuTH has responsibility for ensuring the appropriate regulatory and ethical approvals are in place as required. Due to the expertise within individual sites, the activity of submitting applications to relevant competent authorities will be formally delegated.

## vi. ROLES AND RESPONSIBILITIES OF STUDY MANAGEMENT COMMITEES/GROUPS & INDIVIDUALS

### Study Management Group

The Study Management Group (SMG) consists of all individuals responsible for the day-to-day management of the study. The group will meet on a monthly basis to monitor the conduct and progress of the study, ensure that the protocol is adhered to, and to take appropriate action to safeguard participants and the quality of the study.  This is a low risk observational study that does not require a specific Data Monitoring Committee. Data issues and adverse safety events will be reported to the SMG.

### Study Steering Committee

The Study Steering Committee (SSC) will provide overall supervision of the study. The SSC has an independent Chair and a majority independent representation, including patient representatives. The SSC will monitor study progress and is responsible for making top-level decisions. The SSC will consider and act, as appropriate, upon the recommendations of the Data Monitoring Representative and ethics advisor and ultimately carries the responsibility for deciding whether the study should be stopped on grounds of safety or efficacy. Following each SSC meeting a report will be sent to the sponsor.

## vii. Protocol contributors

The protocol has receieved input from expert individuals involved in the Data Management and the Statistical Analysis work packages.

Scientific Advice from the European Medicines Agency (EMA) has been sought regarding the adequacy of sample size, data collection methods, endpoints, and inclusion/exclusion criteria to facilitate regulatory acceptance of the results. European Federation of Pharmaceutical Industries and Associations (EFPIA) members have contributed through involvement of their experts in the clinical and regulatory aspects of the study conduct.

All regulatory activities will be conducted through a Qualification Task Force (QTF) which includes all regulatory experts in the consortium. The QTF will engage first the EMA Innovation Task Force to explore the possibility to qualify separately the wearable sensors, the analytics software, the digital

mobility outcomes, and to align on required inputs and expectations for the formal Qualification Advice.

**viii. KEY WORDS:** Clinical validation, real-world walking speed, digital mobility assessment, regulatory approved endpoints, EMA approval

## ix. STUDY FLOW CHART

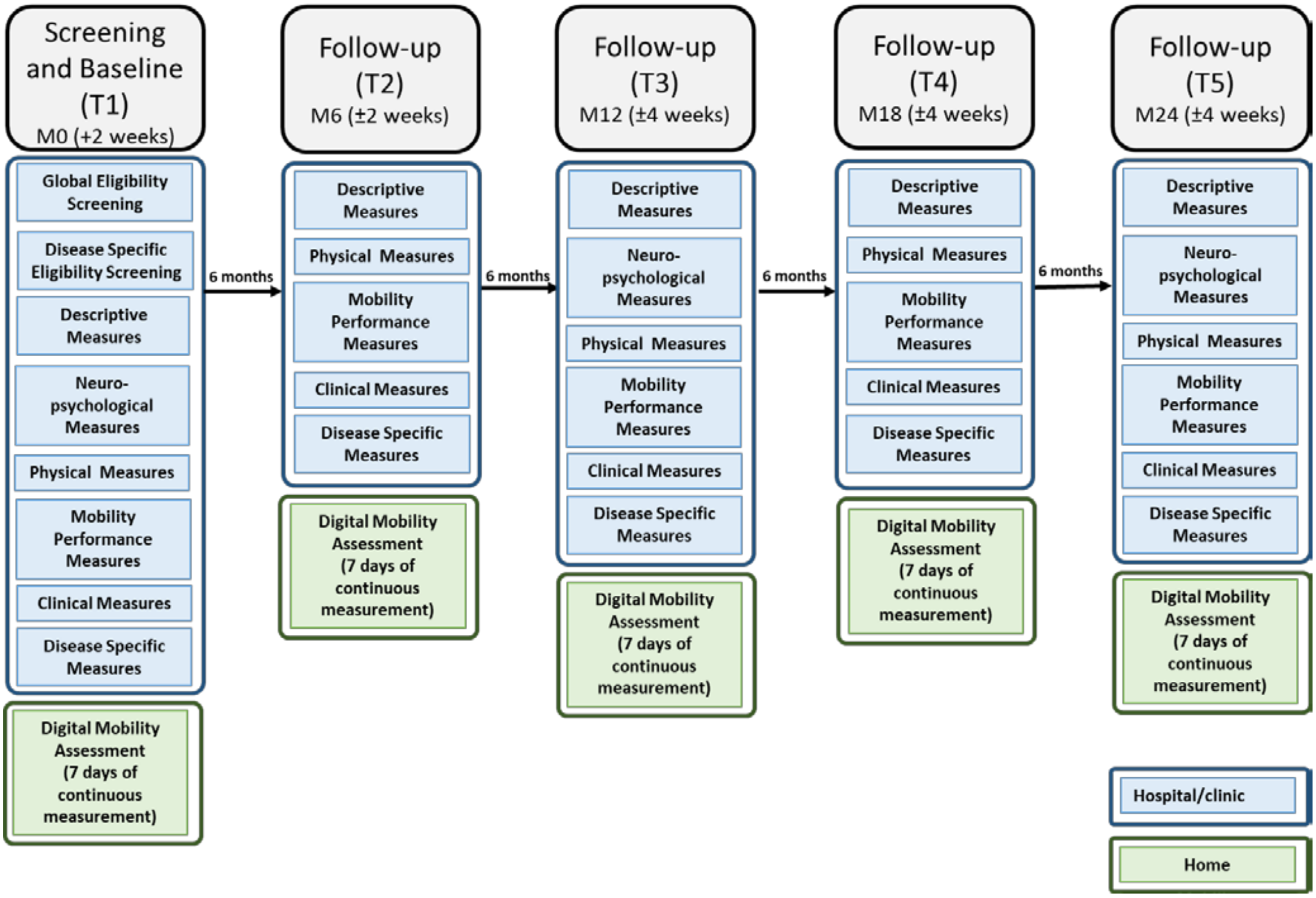


**Figure 1: Study Flow Chart**

# 1 BACKGROUND

The ability to move is a key contributor to "physical, mental and social well-being" which defines health[1]. However, the study of mobility has received little attention, except in diseases characterised by specific mobility dysfunction. The increasing longevity of the world's population together with prolonged survival in many chronic diseases means that more people are suffering from loss of mobility, which in turn is a major determinant of loss of independence. This has a considerable and

growing personal, societal and economic impact. Efforts to mitigate this loss of mobility are an increasing priority and promising interventions are now under investigation. To target mobility loss effectively and thus be able to prevent it, we need valid tools that can detect and measure it. Existing mobility endpoints based on performance, patient self-reporting and one-off assessment are resource intensive and lack sensitivity, which limits therapeutic development and clinical management. A novel approach is needed that is low cost, simple, accurate and capable of use in the real world, including the home and the community. Wearable digital technology (small devices worn on the body that measure movement) has the potential for measuring and monitoring real-world walking speed (RWS) and other digital mobility outcomes (DMOs).

An EU-funded IMI consortium called Mobilise-D aims to develop and implement a digital mobility assessment solution to demonstrate that DMOs can successfully predict relevant clinical outcomes and provide a better, safer and quicker way to arrive at the development of innovative medicines. The first stage of this project is a technical validation of a device-algorithm pair to measure RWS and other DMOs. This study is also an investigation into the usability and acceptability of the device and the data collection methods from the perspective of the participants and researchers. The second stage of the project aims to use this technically validated device-algorithm pair to link DMOs to clinical endpoints for regulatory approval.

The Mobilise-D Clinical Validation study is a longitudinal observational cohort study conducted in ten different countries across 16 different sites. The study will enrol participants from four different disease cohorts; Chronic Obstructive Pulmonary Disease (COPD), Parkinson's disease (PD), Multiple Sclerosis (MS) and Proximal femoral fracture (PFF).

## 2 RATIONALE

Stakeholders from academic and pharmaceutical communities have agreed on the following research priorities:

- the need for validated DMOs to accurately measure mobility in real-life settings
- the need to link DMOs to relevant clinical outcomes across multiple patient cohorts
- the need to obtain regulatory approval to allow the DMOs and DMA to be used in clinical trials and healthcare provision.

Addressing these priorities will enable more efficacious and safer future drug development including early proof of principle, phase II trials and phase III registration trials, and will furthermore improve clinical management.

The four clinical cohorts were selected to maximise variability in mobility difficulties. The aim is to have a generalizable clinical population representing neurodegenerative conditions (PD), respiratory disease (COPD), neuro-inflammatory problems (MS), fall related injuries, osteoporosis, sarcopenia and frailty (PFF). All participants will be followed for 24 months and will be evaluated at five time points. This will allow a deeper insight into long-term mobility trajectories. The 17 sites provide a good geographical representation across Europe. There is diverse representation of age and gender as well as different health care services (in- and outpatient; NHS and non-NHS countries).

## 2.1 Assessment and management of risk

The study is observational, non-interventional and has no known risks to human participants.

# 3 OBJECTIVES AND OUTCOME MEASURES

The general objective is to validate Digital Mobility Outcomes (DMOs) obtained from Mobilise-D algorithms in four chronic conditions – PD, MS, COPD and PFF.

## 3.1 Primary objective

The primary objective is to assess the capacity of DMOs to predict global and disease specific clinical endpoints:

- **Global (all cohorts):** The Late-Life Functional Disability Index (LLFDI).
- **Disease-specific:** fall frequency (PD); fall frequency (MS); occurrence of moderate to severe exacerbations (COPD); and admission to care home (PFF).

## 3.2 Secondary objectives

Secondary objectives are to:

- Assess the capacity of DMOs to predict global and disease specific clinical endpoints (others than those mentioned in the primary objective).
- Assess construct validity of DMOs by describing relationship with general and disease-specific clinically relevant constructs.
- Assess ability of DMOs to detect 24 months change in clinically relevant constructs.
- Estimate the Minimal Important Difference (MID) of DMOs to measure change in disease (worsened or improved) over 24 months.
- To describe real-world walking behaviour with DMO's in patients with PD, MS, COPD and following PFF.

## 3.3 Primary outcome

Primary outcomes are the constructs against which we define predictive ability of the DMOs, specifically:

**Global (all cohorts) Primary Outcome:** Change in the functional component score of the Late-Life Functional Disability Index (LLFDI) during 24 months follow-up.

**Disease Specific Primary Outcomes:**

- PD Cohort: Fall frequency during 24 months follow-up.
- MS Cohort: Fall frequency during 24 months follow-up.
- COPD Cohort: Occurrence of moderate-to-severe COPD exacerbations during the first 12 months of follow-up.

- PFF Cohort: Admission to a care home at six months follow-up.

### 3.4 Secondary outcomes

Secondary outcomes are grouped according to the objective to which they are related.

- Assess **predictive capacity** of DMO's with each assessment as listed below:
  - **Global (all cohorts):**
    - Clinical: mortality, hospital admission, care home admission, falls, changes in medication, changes in functional status (LLFDI disability), changes in health-related quality of life (EQ-5D), comorbidity (FCI Groll), frailty (FI), pain (VAS), fatigue (FACIT).
    - Neuropsychological: changes in cognitive function (D8-MMSE), depression (PHQ-2), fear of falling (Short FES-I).
    - Physical: changes in functional capacity (SPPB), physical capacity (6MWT, timed walking test, TUG, balance), strength (hand grip, 5CRT).
  - **PD Cohort:** changes in disease severity (MDS-UPDRS III and II), changes in physical capacity (mini-BESTest), changes in freezing status.
  - **MS Cohort:** changes during follow-up in disease severity (EDSS), changes in walking ability (T25-FW, MSWS-12).
  - **COPD Cohort:** time to first moderate to severe COPD exacerbation, rate of moderate to severe exacerbations, physical activity experience (C-PPAC).
  - **PFF Cohort:** changes in ability to undertake activities of daily living (BI, NEADL), changes in cognitive impairment (MoCA, CDR).

- Assess **construct validity** of DMO's:
  - **Global (all cohorts)**:
    - Descriptive: Age, anthropometry (depending on DMO).
    - Clinical: Medication, functional status (LLFDI disability), health-related quality of life (EQ-5D), frailty (FI), pain (VAS), fatigue (FACIT).
    - Neuropsychological: Cognitive function (D8-MMSE), depression (PHQ-2), fear of falling (Short FES-I), social isolation and loneliness (UCLA), sleep disturbance.
    - Physical: capacity (SPPB, mean and total distance 6MWT, timed walking test, TUG, balance), strength (hand grip, 5CRT).
  - **PD Cohort:** disease severity and status (MDS-UPDRS total, III and II, Hoehn & Yahr), occurrence and frequency of tremor (MDS-UPDRS III), cognitive impairment (MoCA), physical capacity (mini-BESTest).
  - **MS Cohort:** disease severity (EDSS), walking capacity (T25-FW, MSWS-12).
  - **COPD Cohort:** lung function, history of previous exacerbations, quality of life (CAT), mMRC Dyspnoea, quadriceps muscle force, physical activity experience (C-PPAC).
  - **PFF Cohort:** daily living activities (BI, NEADL), cognitive impairment (MoCA, CDR).

- Assess ability of DMO's to **detect change over 24 months**
  - **Global (all cohorts)**:
    - Global anchor questions.

        - Clinical: Medication, functional status (LLFDI disability), health-related quality of life (EQ-5D), frailty (FI), pain (VAS), fatigue (FACIT).
        - Neuropsychological: cognitive function (D8-MMSE), depression (PHQ-2), fear of falling (Short FES-I).
        - Physical: functional capacity (6MWT), change in strength (hand grip, 5CRT) balance (SPPB- balance component).
    - **PD Cohort:** disease severity (MDS-UPDRS III and II), physical capacity (mini-BESTest).
    - **MS Cohort:** disease severity (EDSS), walking capacity (T25-FW, MSWS-12).
    - **COPD Cohort:** occurrence of exacerbations during follow-up, mMRC Dyspnoea, quadriceps muscle force, physical activity experience (C-PPAC).
    - **PFF Cohort:** ability to undertake activities of daily life (BI, NEADL), cognitive impairment (MoCA, CDR).

- Estimate the Minimum Important Difference of DMOs:
    - **Global:** global anchor questions, LLFDI
    - **PD Cohort:** MDS-UPDRS III and II
    - **MS Cohort:** EDSS, T25-FW, MSWS-12
    - **COPD Cohort:** physical activity experience (C-PPAC), steps/day measured over 7 days, 6MWD, quality of life (CAT).
      **PFF Cohort:** SPPB, LLFDI (Disability component), Upright (standing/walking) time (DMA).

- DMOs to **describe real-world walking** behaviour:
    - Average walking speed over a rectilinear walking bout (WB) [m/s]
    - Metrics extracted per each Walking Bout:
        - Cadence [steps/min] (Rhythm)
        - Step/stride time [s] (Rhythm, variability, asymmetry)
        - Swing/stance time [s] (Rhythm, variability, asymmetry)
        - Single /double support time [s]
        - Stride length [m] (Pace)
        - Turn duration [s]
        - Turn angle [degrees]
        - Turn velocity (mean and peak) [degrees/s]
    - Metrics extracted from all walking bouts (at daily level)
        - Volume of walking: number, length and duration of walking bouts
        - Number of steps per day, walking time, walking/movement intensity, VMU/min
    - Upright time [minutes] (at daily level) including all standing/walking episodes

## 4 STUDY DESIGN

The Mobilise-D Clinical Validation study is a longitudinal (non-interventional) observational cohort study. A total of 2,400 participants across four different disease cohorts will be recruited from 17 clinical sites. Each participant will be followed up every 6 months for a total of 24 months.

## 5 STUDY SETTING

The study will be conducted at 17 different clinical sites across ten different countries (Belgium, France, Germany, Greece, Italy, Spain, UK, Switzerland, Norway, Israel). The clinical sites have been selected based on their pre-existing and sustained expertise in recruiting, assessing and following patients in the study disease. All sites have a tested track record to conduct observational studies and have participated in pharmaceutical and non-pharmaceutical intervention trials. Most sites also have extensive experience with sensor-based assessments of mobility.

## 6 PARTICIPANT ELIGIBILITY CRITERIA

| Group | Inclusion Criteria | Exclusion Criteria |
|---|---|---|
| **All** | • Able to walk 4 meters independently with or without walking aids<br>• Anticipated availability for repeated study visits over 24 months<br>• Ability to consent and comply with any study specific procedures.<br>• Willingness to wear a wearable sensor for mobility monitoring<br>• Able to read and write in first language in the respective country | • Occurrence of any of the following within 3 months prior to informed consent: myocardial infarction, hospitalization for unstable angina, stroke, coronary artery bypass graft (CABG), percutaneous coronary intervention (PCI), implantation of a cardiac resynchronization therapy device (CRTD), active treatment for cancer or other malignant disease, uncontrolled congestive heart disease (NYHA class >3), acute psychosis or major psychiatric disorders or continued substance abuse |
| **PD Cohort** | • Aged 18 or over<br>• Patients with the clinical diagnosis of PD according to the recent criteria of the Movement Disorder Society[2]<br>• Hoehn & Yahr stage I-III | • History consistent with Dementia with Lewy Bodies (DLB), atypical parkinsonian syndromes (including multiple system atrophy or progressive supranuclear palsy, diagnosed according to accepted criteria)<br>• Repeated strokes or stepwise progression of symptoms, leading to a diagnosis of ‘vascular parkinsonism’<br>• Drug-induced Parkinsonism |

| | | |
|---|---|---|
| **MS Cohort** | • Aged 18 or over<br>• A diagnosis of MS based on the revised McDonald's criteria<br>• EDSS score of 3.0-6.5<br>• Clinical evidence of disability worsening over the previous two years | • Clinical relapse within 30 days prior to screening and baseline. |
| **COPD Cohort** | • Aged 18 or over<br>• Diagnosis of COPD (post-bronchodilator forced expiratory volume in the first second ($FEV_1$) to forced vital capacity (FVC) ratio <0.70<br>• Clinical stability, defined as at least 4 weeks after the onset of the last exacerbation<br>• Current or ex-smokers with a smoking history equivalent to at least 10 pack years (1 pack year = 20 cigarettes smoked per day for 1 year) | • Having undergone major lung surgery (e.g. lung transplant)<br>• Current diagnosis of lung cancer<br>• Primary respiratory diseases other than COPD<br>• Substantial limitations in mobility due to factors other than COPD<br>• Lung volume reduction within 6 months before inclusion |
| **PFF Cohort** | • Aged 45 or over<br>• Surgical treatment (fixation or arthroplasty) for a low-energy fracture of the proximal femur (ICD-10 diagnosis S72.0, S72.1, S72.2) as diagnosed on X-rays of the hip and pelvis. Between 3 days and 52 weeks post-surgery | • Not able to walk before treatment of hip fracture |

**Table 1: Global and disease specific eligibility criteria**

# 7 STUDY PROCEDURES

## 7.1 Recruitment

Participants will be recruited from research registers, out-patient and in-patient services. A recruitment poster may be put up in relevant clinic waiting rooms to inform potential participants about the study. GP practices may screen local databases and provide potential participants with contact details for researchers at the local study site.

Patients' medical notes will be screened against the eligibility criteria by a member of the clinical team (e.g. physiotherapists/neurologists/nurse specialists). For participants who are eligible and are interested in taking part, an invitation letter and participant information sheet will be provided. These

documents include the contact details of the research team should they wish for further information regarding the study. Participants must be given sufficient time (according to local guidelines) to read the participant information sheet and to consider whether to take part in the study. A member of the research team will contact each potential participant to discuss the study and to answer any questions. All participants who express an interest in participating in the study will be offered a screening appointment at a mutually convenient time. Approached participants that are found to be ineligible or not interested in participating in the study will be recorded on a pre-screening log. This will record year of birth and the reason for non-participation (if provided).

## 7.2 Screening

All interested potential participants will attend a screening appointment. This will consist of informed consent (section 7.3) and a review of the global and disease specific inclusion and exclusion criteria. A full outline of the recruitment process is illustrated below.

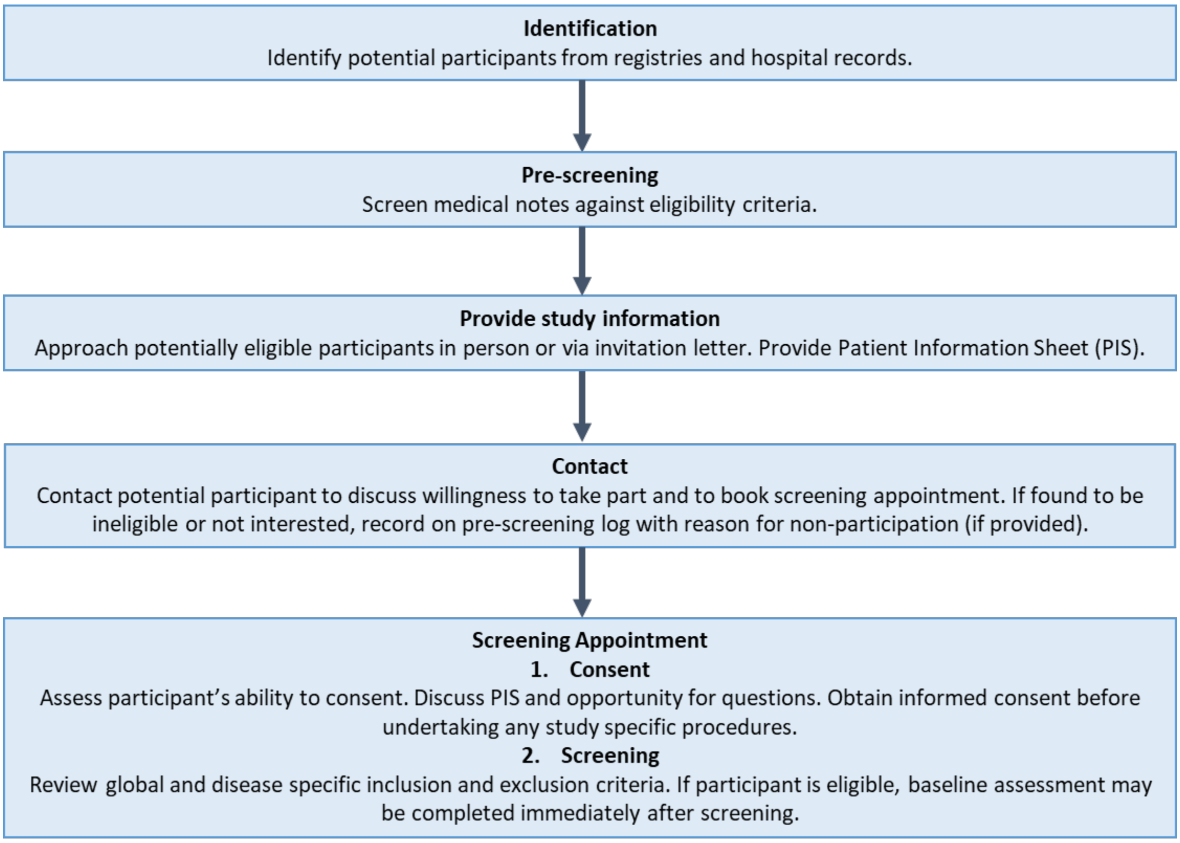


**Figure 2. Flow chart to illustrate full recruitment process.**

## 7.3 Consent

The Principal Investigator (PI) has overall responsibility for the conduct of research at their site, including obtaining informed consent of participants at their site. They must ensure that any person delegated responsibility to obtain informed consent process is duly authorised. Persons delegated to obtain consent must have appropriate training and experience and up-to-date Good Clinical Practice certification.

Informed consent must be obtained prior to the participant undergoing procedures that are specifically for the purposes of the study and are outside of standard routine care at the participating site. The right of a participant to refuse participation without giving reasons must be respected. The researcher must assess that the participant is capable of giving consent for themselves.

The researcher taking consent will confirm that the potential participant has read the participant information sheet before discussing the study further and answering any questions he/she may have. Providing the potential participant agrees to participate they will be asked to sign and date the informed consent form. This will be witnessed by the researcher taking consent, who will also sign and date the form. The informed consent process/discussion will be documented in the participants' medical records. The original consent form will be filed in the site file. A copy will be provided to the participant and a copy will be filed in the participant's medical records according to local requirements.

All participants remain free to withdraw at any time from the study without giving reasons and without prejudicing his/her further treatment and must be provided with a contact point where he/she may obtain further information about the study. Data collected up to the point of withdrawal can only be used after withdrawal if the participant has consented for this. Any intention to utilise such data should be outlined in the consent literature. Where a participant is required to re-consent or new information is required to be provided to a participant it is the responsibility of the PI to ensure this is done in a timely manner.

The PI takes responsibility for ensuring that all vulnerable participants are protected and participate voluntarily in an environment free from coercion or undue influence.

### 7.4 Payment

Participants can receive reimbursement of reasonable expenses, according to local guidelines. Travel expenses may include own vehicle, public transport or taxi.

### 7.5 Study visits

**Baseline Visit:** The baseline (T1) can be completed immediately after screening. If this is not completed as a single visit, the baseline must be completed a maximum of two weeks after the screening visit.

**Follow-up visits**

- **Follow-up (T2)** should be completed six months after the screening visit (±2 weeks).
- **Follow-up (T3)** should be completed 12 months after the screening visit (± 4 weeks).
- **Follow-up (T4)** should be completed 18 months after the screening visit (±4 weeks).
- **Follow-up (T5)** should be completed 24 months after the screening visit (± 4 weeks).

If an exacerbation or other adverse event is experienced during the participant's follow-up visit window, an extended window of up to 4 weeks after the event will be permitted.

If a follow-up visit is not completed within the accepted time window, it should be classed as a missed visit, and the participant should be invited to attend their next follow-up visit.

All follow-up visits may be offered as a home visit if the participant is not able or willing to attend a hospital visit. The reason for conducting the home visit must be documented. Subsequent visits are to be completed in clinic again, unless participants are still unable or unwilling to attend a hospital visit.

## 7.6 Study Assessments

The following assessments will be performed according to the assessment schedule in Appendix 1. A detailed description of each assessment is outlined in the Assessment Manual.

**Descriptive Measures:**

- **General descriptive measures**
  Year of birth, gender, height, weight, shoe size, leg length, education, employment, marital status, living arrangement, overall health status, smoking history, alcohol consumption, ethnicity.
- **Vision (Snellen Chart)**
  A Snellen chart is used as a measure visual acuity at T1.
- **COVID-19 Questionnaire**
  Short questionnaire to capture the COVID-19 history of patients as well as COVID-19 related circumstances. This will be completed at each visit.

**Clinical outcome measures:**

- **Late-Life Functional Disability Index (LLFDI)**
  The LLFDI assesses function and disability in older adults. The functional component (32 items) reflects a person's ability to perform specific actions or activities and the disability component (16 items) reflects a person's ability to perform socially defined life tasks within a typical sociocultural and physical environment. This will be completed at each visit.
- **Mortality**
  The date and reason of death will be recorded from T2 onwards.
- **Care home admission and length of stay**
  Admission to care home will be recorded from T2 onwards.
- **Hospital admission**
  Admission to hospital (for more than 24 hours) and reason of admission within the last 6 months will be recorded.
- **Fall events (occurrence and frequency) and fall related injuries.**
  The number of falls and whether the falls were injurious will be recorded. Twelve month retrospective during T1, six month retrospectively all subsequent visits.
- **Fracture history**
  Number and type of fracture sustained will be recorded. Twelve month retrospective during T1, six month retrospectively all subsequent visits.
- **Medication and non-pharmacological interventions**
  Current medication and non-pharmacological interventions will be recorded at each visit.
- **Blood pressure**

Systolic/diastolic blood pressure measurement (seated position) will be recorded on yearly basis (T1, T3 and T5).

- **Euro-Qol (EQ-5D)**
  The EQ-5D measures quality of life. It consists of two components; health state description and evaluation. This will be completed on yearly basis (T1, T3 and T5).
- **Pain - Visual Analogue Scale (VAS) during rest and walking**
  The VAS measures the amount of pain that a patient feels ranges across a continuum. This will be completed at each visit.
- **Groll Functional Comorbidity Index (FCI Groll).**
  The FCI assesses comorbidity with physical function as the outcome of interest. This will be completed at T1 and T5.
- **Frailty Index (FI)**
  The FI measures frailty five different criteria (shrinking, low physical endurance/energy, low physical activity, weakness and slow walking speed). This will be completed at T1 and T5.
- **Functional Assessment of Chronic Illness Therapy (FACIT) Fatigue scale**
  The FACIT Fatigue Scale measures fatigue during usual daily activities over the past week. This will be completed on yearly basis (T1, T3 and T5).
- **Global Rating of Change**
  Single item question (anchor) on the ease of walking and walking difficulties and possible changes since the last visit to estimate the minimal important difference (MID). Recorded from T2 onwards.
- **Bioelectrical Impedance Analysis (BIA)**
  BIA is a method used for estimating body composition, in particular body fat and muscle mass. Completed at T1 and T5.

**Physical measures (all assessments will be instrumented using a wearable sensor):**

- **Use of mobility aids**
  The use of commonly used walking aids (indoor and outdoor) will be recorded. This will be recorded at each visit.
- **Short Physical Performance Battery (SPPB)**
  The SPPB assesses lower extremity function and mobility. This consists of a static balance task, a five chair-raise test and 4m walk test. This will be completed at each visit.
- **Hand grip strength**
  Hand grip strength is a measure of upper-body skeletal muscle function and is used as a general indicator of frailty. This will be completed on yearly basis (T1, T3 and T5).
- **Timed Up and Go (TUG)**
  The TUG is a common clinical measure used to assess mobility, balance and walking ability in older adults. This will be completed from T2 onwards.
- **Six minute walking test (6MWT)**
  The 6MWT is used to measure functional exercise capacity. The distance in meters covered in 6 minutes is recorded. This will be completed at each visit.
- **Digital Mobility Assessment (DMA)**

The DMA will consist of seven days' unsupervised monitoring of mobility. A wearable sensor will be attached to the participant by the assessor either at the clinic or home visit, and will be worn continuously for at least seven days. Multiple DMO's will be derived from the sensor using validated algorithms and examples include: walking speed, step length, step variability and walking bouts.

**Mobility life space measures:** assessed T2 onwards

- **University of Alabama at Birmingham Life Space Assessment (LSA)**
  The LSA assesses the extent and frequency of movement during the 4 weeks prior to the assessment.
- **Nursing Home Life Space Diameter (NHLSD)**
  The NHLSD assesses the extent and frequency of movement of a nursing home resident in the two weeks prior to assessment.

**Neuropsychological measures:**

- **Short Falls Efficacy Scale International (Short FES-I)**
  The short FES-I is a measure the level of concern about falling during social and physical activities inside and outside the home. This will be completed on yearly basis (T1, T3 and T5).
- **Patient Health Questionnaire (PHQ-2)**
  The PHQ-2 is used to monitor the severity of depression. This will be completed on yearly basis (T1, T3 and T5).
- **Social isolation and loneliness (UCLA Loneliness scale)**
  A simple three item scale to measure social isolation and loneliness. This will be completed on yearly basis (T1, T3 and T5).
- **Mini-Mental State Examination Short version (SMMSE)**
  The SMMSE is a measure cognitive impairment and to predict dementia. This will be completed at T1 and T5.

**Disease Specific Assessments:**

**PD Cohort:**

- **Movement Disorder Society Unified Parkinson's Disease Rating Scale (MDS-UPDRS)**
  The MDS-UPDRS describes disease progression. It is separated into four different domains including cognitive function, behaviour and mood, activities of daily living (ADL) and motor examination. Part III (motor examination) will be completed at each follow-up visit and a full version will be completed on a yearly basis (T1, T3 and T5).
- **Mini balance Evaluation Systems Test (Mini BESTest)**
  The Mini BESTest is a measure to assess dynamic balance. Completed on yearly basis (T1, T3 and T5).
- **New Freezing of Gait Questionnaire (NFOGQ)**
  The NFOGQ is a tool to detect and evaluate the impact and severity of freezing of gait. Full assessment completed baseline, Q1 only from T2 onwards.

- **Montreal Cognitive Assessment (MoCA)**
  The MoCA is a measure of cognitive impairment. It assesses different cognitive domains: attention and concentration, executive functions, memory, language, visuo-constructional skills, conceptual thinking, calculations, and orientation. Completed on yearly basis (T1, T3 and T5).

**MS Cohort:**

- **Modified Fatigue Impact Scale (MFIS)**
  A 21 item scale derived from patient interview which covers the impact of fatigue of patients' lives. Completed at all visits.
- **The Multiple Sclerosis Functional Composite (MSFC)**
  The MSFC is a measure of MS in three key clinical dimensions: leg function and ambulation (Timed 25-Foot Walk), arm and hand function (9-Hole Peg Test), and cognitive function (Paced Auditory Serial Addition Test). Completed at all visits.
- **Expanded Disability Status Scale (EDSS)**
  The EDSS is an ordinal clinical rating scale ranging from 0 (normal neurologic examination) to 10 (death due to MS) in half-point increment. It is used to quantify disability in MS. Completed at all visits.
- **Patient Determined Disease Steps (PDDS) scale**
  The PDDS is a patient reported measure of disability in MS. Completed at all visits.
- **Multiple sclerosis walking scale-12 (MSWS-12)**
  The MSWS-12 is a patient reported measure of impact of MS on walking ability. Completed at all visits.
- **Symbol Digit Modalities Test (SDMT)**
  The SDMT is used to assess severity of cognitive dysfunction. Completed at all visits.
- **Low-contrast letter acuity (LCLA)**
  A series of seven contrast levels for accurate and precise vision testing. Completed at all visits.

**COPD Cohort:**

- **Spirometry**
  Spirometry on usual medication will be used to measure lung function (FEV-1, FVC and FEV1/FVC ratio). Completed at all visits.
- **Exacerbations**
  Occurrence of moderate-to-severe COPD exacerbations due to COPD will be recorded at all visits.
- **2nd Six minute walking test (6MWT)**
  A second 6MWT will be undertaken at every study visit. The best of two tests will be used as COPD specific outcome, the first 6MWT will be used as general outcome.
- **Smoking and E-cigarette use**
  Any changes in smoking habits and/or e-cigarette use will be recorded from T2 onwards.
- **COPD Assessment Test (CAT)**
  The CAT is a patient reported measure used to quantify the impact of COPD on overall health. Completed on yearly basis (T1, T3 and T5).
- **Modified Medical Research Council (mMRC) Dyspnoea Scale**

The mMRC is used to measure the effect of breathlessness on daily activities. Completed at all visits.

- **PROactive Physical Activity in COPD (clinical visit) (C-PPAC)**
  The C-PPAC is a measure of physical activity experience in COPD patients. Completed at all visits.
- **Isometric Quadriceps muscle force (QF)**
  The isometric quadriceps test is a measure of muscle strength. Completed on yearly basis (T1, T3 and T5).
- **Oxygen saturation**
  Oxygen saturation at rest will be recorded on yearly basis (T1, T3 and T5).

**PFF Cohort:**

- **Descriptive information regarding fracture and treatment**
  Injury date, time, time of admission, time and date of surgery, type of anaesthesia will be recorded, fracture type and surgery method will also be collected at T1.
- **American Society of Anaesthesiologists Classification (ASA)**
  Scoring system for the evaluation of the patients' health and comorbidities before an operative procedure will be collected at T1.
- **Barthel Index (BI)**
  Personal ADLs will be measured by the Barthel Index, an ordinal scale used ranging from 0-20 where a high score suggests increased independence. Assessment at baseline (T1) will capture pre-fracture status, possibly collected using a proxy. Repeated on yearly basis (T3 and T5).
- **Nottingham Extended Activities of Daily Living (NEADL)**
  Instrumental ADLs will be measured by use of the NEADL, with scores ranging from 0-66 with a higher score indicating better ability to undertake instrumental ADLs. Assessment at baseline (T1) will capture pre-fracture status, possibly collected using a proxy. Repeated on yearly basis (T3 and T5).
- **4AT Delirium scale**
  Assessment of delirium during hospital admission (first and/or second postoperative day) will be undertaken during baseline visit of acute participants.
- **Clinical Dementia Rating Scale (CDR)**
  Assessment of cognitive status pre-fracture and on yearly basis (T1, T3 and T5).
- **Montreal Cognitive Assessment (MoCA)**
  The MoCA is a measure of cognitive impairment. Completed at T2 and T5.
- **Subjective Hearing Impairment**
  Two questions to assess subjective hearing impairment. Completed at baseline (T1) and T5.

### 7.7 Diaries

MS and PD participants will be asked to complete a daily falls diary. Participants will be provided with pre-paid envelopes to return the diaries on a monthly basis. If diaries are not returned, participants will be contacted by telephone. COPD participants will be asked to complete a daily exacerbation diary. This will record medication changes, hospitalisations, unplanned doctor's visit and falls. Participants will return the completed diaries at each visit, and will be sent monthly reminders to complete these.

## 7.8 Environmental Data Capture

Environmental factors which may impact DMOs will be collected. These include variables that are considered core elements for weather characterisation in the National Oceanic and Atmospheric Administration (e.g. precipitation and snowfall) and those that have been suggested as relevant for mobility (e.g. cloud cover, sunshine duration and wind). This data will be collected at the level of closest city/town of residence and on a daily basis (for the seven days the sensor is worn by the participant).

## 7.9 Withdrawal from study

All participants remain free to withdraw at any time from the study without giving reasons and without prejudicing his/her further treatment and must be provided with a contact point where he/she may obtain further information about the study.

## 7.10 End of study

The end of study will be defined date of the last visit/data item of the last participant undergoing the study.

# 8 SAFETY MONITORING

This is a non-interventional observational study and therefore no adverse event reporting is required. Serious Adverse Events (SAEs) must be reported if they are related to the study (ie they resulted from participation in any of the research assessments). Expected events (such as acute exacerbations of COPD leading to hospital admission) are not considered SAEs.

SAEs are untoward medical occurrences that:

- results in death
- is life-threatening
- requires inpatient hospitalization or causes prolongation of existing hospitalisation
- results in persistent or significant disability/incapacity
- may have caused a congenital anomaly/birth defect

An SAE form should be completed and returned to the Project Administrator within 24 hours of the site's awareness of the event. This form must be reviewed and signed off by the site PI or Co-I. The CI will be informed immediately of any SAE and will determine the seriousness and causality in conjunction with the study procedures. If it is deemed that the SAE is related to the study procedures, a report will be submitted to the REC using the Non-CTIMP safety report to REC form. These should be sent within 15 days of the CI becoming aware of the event.

# 9 STATISTICS AND DATA ANALYSIS

## 9.1 Sample size calculation

**Global primary outcome (all disease cohorts): Changes in Late-Life Functional Disability Index (LLFDI) in 24 months**

We hypothesise that baseline real-world walking speed is associated with the 24 months changes in the functional component of the Late-Life-Functional-Disability-Index (LLFDI, score: 0-100). We assume (i) a minimal detectable change with 95% confidence of 3.41 in LLFDI functional component[4], which is able to detect clinically relevant intervention effects[5], (ii) a standard deviation of the mean change in LLFDI functional component of 30.1 points (derived from ref Beauchamp), and (iii) a standard deviation of 0.29 m/s for real-world walking speed[6]. We further assume that a difference of 0.1 m/s in real-world walking speed between two patients is clinically relevant and measurable. Based on a linear regression analysis a sample size of 67 subjects would allow to identify as statistically significant a change of 3.41 points within 24 months in LLFDI functional component per 0.1 m/s in baseline real-world walking speed with a power of 80% and an alpha error of 0.05. Including an expected drop-out rate of 20% the final sample size would be n=81. The available sample size of 2400 patients exceeds this requirement and therefore allows to test the study hypothesis with sufficient power.

**PD: Fall frequency in 24 months**

Reduced supervised gait speed has been associated with increased fall risk in patients with PD[7]. We hypothesise that real-world walking speed is associated with fall frequency during 24 months follow-up. Assuming: (i) a standard deviation of real-world walking speed of 0.11 or higher based on previous literature[8,9] and own unpublished data, (ii) a 2-y rate of falls of 1.6 or higher based on previous literature[9] and own unpublished data, and (iii) a proportion of lost to follow up during 24 months up to 20%, based on own experience with patients of similar PD severity and projects of similar burden, a recruitment of 600 patients would allow to recognize as statistically significant a coefficient of 1 for a 1 m/s decline in walking speed (as estimated in own unpublished several sources of data between real-world walking speed and falls rate), with a power of 90% and an alpha error of 0.05 using a Poisson regression model.

**MS: Fall frequency in 24 months**

We hypothesise that real-world walking speed is associated with fall frequency during 24 months follow-up. Assuming (i) a standard deviation of real-world walking speed of 0.13 or higher based on previous literature[10] and own unpublished data, (ii) a 2-y rate of falls of 5 or higher based on previous literature[11,12] and own clinical experience, and (iii) a proportion of lost to follow up during 24 months up to 10%, based on own research experience with patients of similar severity, a recruitment of 600 patients would allow to recognize as statistically significant a coefficient of 0.5 or higher for a 1 m/s decline in walking speed (as estimated in own unpublished several sources of data between real-world walking speed and falls rate), with a power of 90% and an alpha error of 0.05 using a Poisson regression model.

**COPD: Occurrence of exacerbations in 12 months**

We hypothesise that real word walking speed is associated with the occurrence of COPD exacerbations in stable patients during 12 months of follow-up. Assuming: (i) a standard deviation of real-world walking speed of 0.115 or higher based on previous literature (Klenk 2016, PLoS One) and own unpublished data, (ii) a proportion of exacerbations up to 0.6 during 12 months based on previous literature and own unpublished data of COPD patients from similar severity from same recruitment sites[13,14,15], (iii) a proportion of lost to follow up during 12 months up to 30%, based on previous literature and own unpublished data of COPD patients from similar severity

from same recruitment sites[13,14,15], a recruitment of 600 COPD patients would allow to recognize as statistically significant an odds ratio of 1.43 for a 0.1 m/s decline in walking speed (as observed between 4 meters gait speed at hospital discharge and further risk of readmission[16]), with a power of 90% and an alpha error of 0.05 using a logistic regression model.

**PFF: Occurrence of Care Home Admission in 6 months**
We hypothesise that real word walking speed is associated with the occurrence care home admission within the next 6 months after recruitment in hip fracture patients. Assuming: (i) a ratio of low RWS vs normal/high RWS of 1:2 based on clinical experience and own unpublished data (D6.1; TVS 2.5h acquisitions), (ii) a 6-m proportion of admissions to care home of 10 to 30% based on previous literature[17,18], (iii) a proportion of lost to follow up during 6 months up to 30%, based on previous literature[17,18], a recruitment of 572 patients (≈600) would allow to recognize as statistically significant an odds ratio of 2.55 in the risk of admission on slow RWS vs normal/high RWS[19], with a power of 90% and an alpha error of 0.05 using a logistic regression model.

## 9.2 Planned recruitment

Approximately 2,400 participants are to be recruited across 17 sites over a period of 12 months. The recruitment totals for each site is outlined in Table 2. Given the timeframe of the study recruitment will be monitored on a monthly basis. Should any site encounter recruitment difficulties, other sites as named in Table 2 below have the capacity to recruit additional participants for each cohort as well as to recruit additional cohorts if necessary.

| Partner | Site (s) | Cohort | Recruitment aim |
|---|---|---|---|
| Katholieke Universiteit Leuven, Belgium | University Hospitals Leuven | COPD | 140 |
| Institut De Salut Global Barcelona, Spain | Hospital del Mar, Barcelona<br>Hospital Clínic, Barcelona<br>Hospital de Viladecans, Viladecans<br>Hospital Germans Trias i Pujol, Badalona | COPD | 140 |
| Pneumologisches Forschungsinstitut an der LungenClinic Grosshansdorf GmbH, Germany | Pneumologisches Forschungsinstitut an der LungenClinic Grosshansdorf GmbH | COPD | 140 |
| Universität Zürich, Switzerland | Epidemiology Institute, University of Zurich<br>Klinik Barmelweid | COPD | 40 |

| | | | |
|---|---|---|---|
| | Zuercher RehaZentrum Wald<br>Berner Reha Zentrum, Heiligenschwendi | | |
| Imperial College of Science, Technology and Medicine, United Kingdom | Guys and St Thomas' NHS Foundation Trust | COPD | 40 |
| University of Northumbria at Newcastle, United Kingdom | Thorax Foundation, Athens | COPD | 50 |
| University of Northumbria at Newcastle, United Kingdom | University of Northumbria at Newcastle, United Kingdom | COPD | 50 |
| Christian-Albrechts-Universität zu Kiel, Germany | University Hospital Schleswig-Holstein, Campus Kiel | PD | 170 |
| Medical Research Foundation and Infrastructure Development Health Services, Israel | Tel Aviv Medical Center, | PD | 170 |
| Universitätsklinikum Erlangen, Germany | University Hospital Erlangen | PD | 160 |
| University of Newcastle upon Tyne, United Kingdom | The Newcastle upon Tyne Hospitals NHS Foundation Trust, UK | PD | 50 |
| Katholieke Universiteit Leuven, Belgium | University Hospitals Leuven; Posture & Movement Analysis Laboratory Leuven | PD | 50 |
| Norwegian University of Science and Technology, Trondheim, Norway | St. Olavs hospital, Trondheim University Hospital | PFF | 250 |
| Robert Bosch Gesellschaft fuer Medizinische Forschung, Germany | Robert Bosch Krankenhaus | PFF | 200 |
| Centre Hospitalier Universitaire de Montpellier, France | Fracture Liaison Service of CHUM | PFF | 150 |

| | | | | |
|---|---|---|---|---|
| Università Vita-Salute San Raffaele, Italy | San Raffaele Hospital | MS | 300 | |
| The University of Sheffield, United Kingdom | Sheffield Teaching Hospitals NHS Foundation Trust | MS | 300 | |
| **Total** | | | | 2,400 |

**Table 2: Recruitment plan**

## 9.3 Statistical analysis plan

### 9.3.1 Analysis plans and datasets

The statistical analysis for the clinical validation of the digital mobility outcomes (DMOs) obtained from Mobilise-D algorithm will follow a step-wise procedure and will be pre-specified in the Statistical Analysis Plan. Briefly it will include the definition of analysis sets, details on data edition (including derivation of new variables), handling of missing data and statistical analysis (including prioritisation of outcomes).

### 9.3.2 Descriptive analysis

Main characteristics of patients, including detailed description of COAs and DMOs, will be done by number and percentage for categorical variables, mean and standard deviation for continuous variables with normal distribution, and median and percentiles 25th-75th for continuous variables with non-normal distribution.

### 9.3.3 Validation analyses

Separate analysis for each cohort/disease will be performed with the corresponding outcomes. Analysis that combine all or some of the cohorts will be performed for outcomes that are common to these cohorts.

**Primary objective – global outcome**

We will test predictive capacity of DMOs against the change during 24 months in the LLFDI functional component, combining standard with hypothesis-free machine learning approaches. Analyses will be conducted pooling the four disease cohorts.

First, as a standard approach, we will assess the association between each DMO (baseline levels) and LLFDI functional score, using univariate and multivariable regression adjusting for confounders. Non-linear associations will be tested using generalised additive models and appropriate transformation of variables will be done consequently. The areas under receiver operating characteristic (ROC) curves measuring DMOs for the prediction of binary outcomes (e.g, hospitalization [yes, no]) will be calculated and displayed graphically for each DMO. Sensitivity, specificity, and accuracy and their corresponding 95% CI for each DMO will be calculated for selected critical cutoffs on the empirical ROC (all observed values). Formal statistical testing of the different DMOs with respect to sensitivity, specificity, accuracy and areas under ROC will be provided.

Secondary analysis will include:

- Use of DMO changes over time as predictors.
- Use of global (all disease cohorts) secondary outcomes (see section 3.4) as outcome variables using the appropriate type of regression models depending on the outcome distribution.

Sensitivity analysis will include:

- Additionally adjusting for baseline levels of LLFDI functional score to account for horse racing effect.
- Meta-analysis by study site to account for heterogeneity in relation to diverse geographic areas (at the country level).

Second, we will use multivariate approaches to study the capacity of combinations and patterns of DMOs to predict changes in LLFDI functional score. These models will use data reduction strategies if there is the need to eliminate redundant input variables and/or avoid correlation between them.

**Primary objective – disease-specific outcomes**

Analysis by disease will be performed using same methods as described above for the corresponding outcomes: frequency of falls during 24 months of follow-up (in PD and MS), proportion of patients with moderate/severe COPD exacerbations during 12 months of follow-up (in COPD), and proportion of patients admitted to care home at 6 months (for PFF), combining standard with hypothesis-free machine learning approaches. All analyses will be conducted by disease cohort.

**Construct validity**

Construct validity will be evaluated by convergent, known-groups and discriminant validity.

To assess convergent validity we will build, prior to analysis, a matrix of expected correlations between each DMO and potentially related constructs (global, or disease-specific, see section 3.4). Then we will estimate the Spearman correlation between DMOs and selected constructs, e.g, correlation between walking speed and SPPB total (in all patients), MDS-UPDRS III (in PD), EDSS (in MS), 6MWT distance (in COPD), no extra measure will be applied for PFF.

To assess known-groups validity, we will assess the distribution of DMOs across groups of defined by known relevant related constructs (see section 3.4), e.g., distribution of real walking speed by age groups (in all patients), Hoehn & Yahr groups (in PD), EDSS (in MS), mMRC dyspnea scale groups (in COPD) or long-term care needs care status and cognitive impairment status (in PFF).

To assess discriminant validity we will build, prior to analysis, a matrix of expected correlations between each DMO and potentially unrelated constructs (global, or disease-specific, see section 3.4). Then we will estimate the Spearman correlation between DMOs and selected constructs, e.g, correlation between walking speed and tremor (in PD), visual impairment (in MS), diastolic blood pressure (in COPD) or arterial hypertension and hearing loss (in PFF).

Secondary analysis will include:

- Stratification of convergent validity analysis by sex, age group, geographic area, disease severity and sarcopenia status.

### Ability to detect change

The ability to detect change of each DMO will be tested against clinically meaningful changes defined as changes in predefined global (all disease) and disease-specific outcomes (see section 3.4).

We will first define groups according to clinically meaningful changes, e.g., global anchor question (self-reported improvement/no change/worsening in DMOs) (in all patients), changes >/< the minimal important difference for MDS-UPDRS III (in PD), changes >/< the minimal important difference for EDSS (in MS), changes >/< the minimal important difference for occurrence of exacerbations (in COPD), and changes >/< the minimal important difference for SPPB score, Barthel Index and LLFDI (disability component) (in PFF).

Then we will assess the distribution of 24-months changes and standardised response mean of each DMO according to the groups previously defined.

### Definition of Minimal Important Difference (MID)

For each DMO and disease, we will estimate the minimal important difference (MID) that would likely be important from the patient's or clinician's perspective by combining anchor- and distribution-based methods.

We will use the following anchors: global anchor questions and LLFDI total score (in all patients), MDS-UPDRS III and II (in PD), T25-FW (in MS), physical activity experience (C-PPAC) and 6MWD (in COPD), SPPB score, Barthel Index and LLFDI (disability component) (in PFF). We will test the correlation between 24 months changes in anchors and 24-months changes in DMOs. Only for the pairs anchor-DMO that exhibited correlations>|0.3|, we will calculate the mean difference (final – baseline) in DMO in patients that changed >/< of MID of the anchor.

For the distribution-based methods, we will use the Cohen's effect size, the empirical rule effect size and, if test-retest results are available, the standard error of measurement.

## 10 DATA MANAGEMENT

All aspects of data management in this study will adhere to guiding principles that research data are findable, accessible, interoperable and reusable (FAIR)[20], as well as being attributable, legible, contemporaneous, original and accurate (ALCOA)[21]. Furthermore, all research will be carried out in compliance with appropriate laws, rules, regulations and guidelines applicable to the collection, use, handling, disposal and processing of personal data. In particular research will adhere to the provisions set out in.

- General Data Protection Regulation (GDPR)[22]
- Directive 2006/24/EC of 15 March 2006 on the retention of data generated or processed in connection with the provision of publicly available electronic communication services or of public communications networks[23]

### 10.1 Data collection tools and source document identification

Participant data will be collected in a coded, de-identified manner, using electronic data capture as a default option. Data will either be entered into the Mobilise-D data management platform directly, or via third party platforms (provided by technical partners ERT and McRoberts). The proposed data flow model is illustrated in Figure 3. Where electronic data capture is not feasible, paper case report forms will be used. Data from paper forms will be transcribed onto the Mobilise-D database and the signed paper forms will be scanned and uploaded to the platform. The Mobilise-D data management platform will be created within the e-Science Central, which is an open source cloud based platform designed to provide secure ingestion, storage, sharing and analysis capability for scientific studies (www.esciencecentral.co.uk). The Mobilise-D e-Science Central platform will be implemented using Amazon Web Services (AWS) located within the European Union.

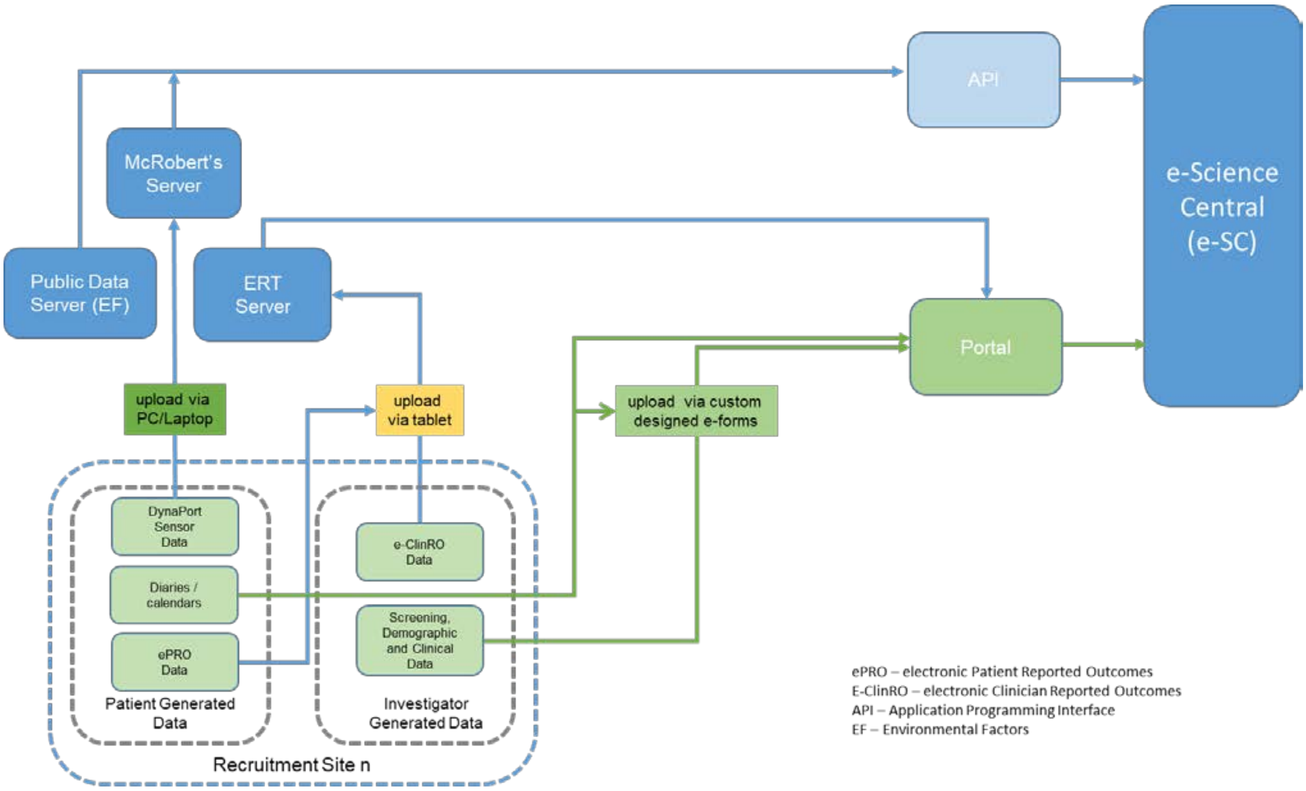


**Figure 3: Mobilise-D Clinical Validation Study – Data Flow Model**

Paper diary data will need to be uploaded on the Mobilise-D database by the clinical investigators at each site.

All recruitment sites will keep original records of all signed consent forms, study key codes, and any other paper forms or samples that are collected at source, under secure conditions at the site or origin

until the study has been completed and the database has been locked. After this point, these documents can be purged or archived for a further period, depending on local requirements.

### 10.2 Data handling and record keeping

A detailed description of the data collection and management, including mechanisms to ensure data quality, completeness and integrity, can be found in the Data Management Plan.

Accumulating data will undergo central checks and data management reports will be generated for resolution of any issues on a monthly basis. Study sites should aim to enter data within 7 days of a study visit. Investigators and research staff at each site will ensure the accuracy, completeness, and timeliness of the data recorded and of the provision of answers to data queries.

The Data Management Plan will describe the methods used to collect, check, validate and process clinical data in detail. It will also clarify the roles and responsibilities for the different functions and personnel involved in the data management process. The Data Management Plan will also describe the data flow and timelines within the study. Quality control procedures will be applied to each stage of data handling to ensure that all data are reliable and have been processed correctly.

When all data have been coded, validated, and locked, clean file will be declared and the final database will be locked.

### 10.3 Access to Data

Direct access will be granted to authorised representatives from the Sponsor, host institution and the regulatory authorities to permit study-related monitoring, audits and inspections- in line with participant consent.

### 10.4 Archiving

Clinical sites will be responsible to archiving all study documents for a period of time that is in keeping with institutional or national guidelines that pertain to that site. Destruction of documentation should be notified to the Sponsor.

## 11 QUALITY CONTROL, MONITORING, AUDIT & INSPECTION

Remote or on-site visits will be conducted at all participating clinical sites prior to the study start. All staff members involved in the study will receive extensive training including a skills test.

A Study Monitoring Plan will be agreed upon by the Study Management Group. This will be dependent on a documented risk assessment of the study. All monitoring will be conducted remotely with a focus on safety monitoring (SAE collection), protocol deviation collection, and source document verification of primary endpoints (deaths, falls, hospitalizations, etc.). A central data management team will undertake continuous data monitoring to ensure completeness and accuracy of clinical and digital data. The flow of screening, recruitment and follow-up of cohorts will be monitored on a monthly basis.

# 12 ETHICAL AND REGULATORY CONSIDERATIONS

## 12.1 Research Ethics Committee (REC) review & reports

Each clinical centre is responsible for the submission and approval of the study protocol to the relevant local ethical committees.

Before the start of the study, a favourable opinion will be sought from the local research ethics committee (REC) for the study protocol, informed consent forms and other relevant documents. Substantial amendments that require review by local REC will not be implemented until that review has been completed and mechanisms are in place to implement at site. All correspondence with the local REC should be retained and sent to the sponsor.

The Chief Investigators is responsible for producing the annual reports as required and to notify the REC of the end of the study. If the study is ended prematurely, the Chief Investigator will notify the REC, including the reasons for the premature termination. Within one year after the end of the study, the Chief Investigator will submit a final report with the results, including any publications/abstracts, to the REC.

Investigators at non-UK sites will responsible for providing the required documentation to their own local ethics committees.

## 12.2 Peer review

The second stage of Mobilise-D proposal (which included the overall study design of the Clinical Validation Study) was reviewed by a panel of independent experts from the EU which included scientific and ethical review.

## 12.3 Patient and Public Involvement and Engagement

The study has been reviewed by VOICE, an international organisation comprised of patients, carers and members of the public. Members contribute insights, experience, ideas and vision to drive innovation on ageing and improve health research. The outcomes of this review have been fed into the design and conduct of the study. The Patient Information Sheet has been reviewed by UK PD and COPD patient groups and updated accordingly. The study assessments have been piloted at all sites.

The project Stakeholder Board will ensure the views of recipients and providers of healthcare are taken into account during the implementation of the project.

## 12.4 Regulatory Compliance

The study will be conducted according to the declaration of Helsinki, Good Clinical Practise (GCP) standards and The European Code of Conduct for Research Integrity[24].

All wearable sensors used in the study have a CE marking can be assumed to be within class I (low-risk potential).The study should therefore be classified as non-significant risk medical device study (Directive 93/42/EEC, Appendix IX). The Medicines and Healthcare products Regulatory Agency have confirmed that regulation of the study is not required. The ultimate classification will be dependent on the decision of local IRBs. The study must be approved by the competent authorities and by the IRB in

the member state where the study will be conducted (Belgium, France, Germany, Greece, Italy, Spain, UK; Switzerland, Norway, Israel). Special attention is given to the compliance and compatibility of EU regulations regarding local regulatory requirements outside EU (e.g. in Israel, Switzerland, Norway and UK). National and federal state requirements (such as state IRB) are mandated.

## 12.5 Protocol compliance

The Principal Investigator (or an appropriate member of the research team) at each site is responsible for reporting protocol deviations/violations. The Study Management Group are responsible for reporting suspected deviations, violations, and serious breaches of the protocol and/or GCP to the Sponsor. The Newcastle Joint Research Office Governance Team will maintain responsibility for the assessment of protocol deviations, violations and serious breaches on behalf of Sponsor, and for ensuring appropriate CAPAs are implemented. The Governance Team are also responsible for reporting Serious Breaches to the REC.

## 12.6 Data protection and patient confidentiality

All data generated will be stored in encrypted and password-locked files with SOA protection measures. Transmission of information via electronic means will be performed using encrypted data files. Disclosure of information from the study to third parties will be limited to those, undertaking legitimate peer review of the medical, scientific and ethical aspects of the study, so that consent can be obtained and customary medical care can be provided. Participant confidentiality and welfare will always be maintained as the highest priority. Anyone with access to data, including the investigator, is subject to professional secrecy during and after the project. All steps will be performed according to GDPR for the European Union.

Data protection and security is described in detail in the Data Management Plan. All data will be de-identified at point of capture. No identifiers will ever be stored alongside any participants' data. Upon entering the study a unique code will be created for each study participant. The study key code, which has details of participants' names and study codes, will be maintained in paper format at each recruitment site. This key code will be kept under secure conditions at each site, as per local guidelines. The minimum level of security will entail the key code being stored in a locked filing cabinet in a locked office. The key-code will be destroyed once database is locked and local guidelines permit – will be maintained for defined period (e.g. 5 years) in sites where this is required.

Only de-identified data will be ingested to Mobilise-D platform, where it will be stored in a secure server. All data will be integrated on the platform using the unique code that is created for each study participant and the file nomenclature system outlined above. We will implement a 'Privacy by Design' protocol on the Mobilise-D platform. This will incorporate application of technical anonymization protocols to render the data to anonymous prior to it being stored in the data warehouse. There will be a very well defined access and governance model in place to ensure that access to the source (de-identified data) is limited to a small core group. Wider access is only available for the anonymised dataset.

## 12.7 Indemnity

Indemnity to meet the potential legal liability of the sponsor for harm to participants arising from the management and conduct of the research will be provided by the NHS indemnity scheme (for UK sites).

Indemnity to meet the potential legal liability of the sponsor or employers for harm to participants arising from the design of the research will be provided by Newcastle University.

Non-UK sites will be responsible for their own local indemnity arrangements.

### 12.8 Access to the final study dataset

We plan to maintain the full anonymised dataset on the Mobilise-D platform indefinitely as part of our commitment to adhere to the open data policy. Once the study is completed the fully anonymised dataset will be made available to the wider research community for secondary research purposes. Participants must give explicit consent for this use. Source data will be maintained at local site of capture in de-identified manner for period of time stipulated by local ethics committee (normally 5 years). Once this period of time has elapsed the original de-identified dataset, and the study key code, will be destroyed.

## 13 DISSEMINIATION POLICY

### 13.1 Dissemination policy

Dissemination of project results is crucially important to reach a long-lasting impact. Mobilise-D has multiple measures in place to maximise dissemination of the results of the clinical validation study, emphasising a stakeholder-driven dissemination strategy and an Open Access policy. The study will be listed on the ISRCTN registry and included on the NIHR Clinical Research Network Portfolio. Relevant stakeholders for Mobilise-D include the scientific community; patients and patient organisations; health care professionals and public health authorities; pharmaceutical and associated industries; regulatory bodies; and the general public. Scientific dissemination will take place through peer-reviewed publications in scientific journals and presentations at scientific conferences, addressing the main objectives of the study in comprehensive primary papers across all included cohorts, as well as consisting of secondary papers focusing on specific cohorts and sub-questions. Furthermore, scientific dissemination will take place through training of early career researchers through direct involvement in the clinical validation study as PhD students and post-doctoral students, as well as through a Mobilise-D summer school targeting students and young professionals in e.g. (bio-)engineering and medical sciences. The results of the clinical validation study will also be disseminated through popular-science and professional publications in a variety of trade journals and magazines. The wider audience will also be kept appraised of the study results through the Mobilise-D website, social media, newsletters, press releases and project videos. In addition, participants in the clinical validation study will receive feedback in the form of newsletters targeted expressly at them.

### 13.2 Authorship eligibility guidelines and any intended use of professional writers

The clinical validation study is expected to result in several primary papers and a wide variety of secondary papers. An overview over all planned publications will be maintained, with an explicit aim to ensure that all partners involved in the project have equal possibility for authorship. Furthermore, emphasis will be placed on ensuring that early career partners in particular will have the opportunity to benefit from their contribution to the study. All partners participating actively in the study are eligible authors. Authorship on publications and presentations will adhere to the ethical guidelines for

authorship on scientific output as recommended by the ICMJE (International Committee of Medical Journal Editors). The ICMJE outlined the following criteria for authorship:

- Substantial contributions to the conception or design of the work; or the acquisition, analysis, or interpretation of data for the work; AND
- Drafting the work or revising it critically for important intellectual content; AND
- Final approval of the version to be published; AND
- Agreement to be accountable for all aspects of the work in ensuring that questions related to the accuracy or integrity of any part of the work are appropriately investigated and resolved.

The use of professional writers is deemed not to be necessary for the publications resulting from the clinical validation study given the outstanding publication expertise in the Mobilise-D consortium.

## 14 REFERENCES

1. World Health Organization, et al. Mental health atlas 2005: World Health Organization, 2005.
2. Postuma et al.- MDS clinical diagnostic criteria for Parkinson's disease. Mov Disord. 2015 Oct;30(12):1591-601. doi: 10.1002/mds.26424.
3. Healy BC, Engler D, Glanz B, Musallam A, Chitnis T. Assessment of definitions of sustained disease progression in relapsing-remitting multiple sclerosis. *Mult Scler Int*. 2013;2013:189624
4. Beauchamp MK, Jette AM, Ward RE, Kurlinski LA, Kiely D, Latham NK, Bean JF. Predictive Validity and Responsiveness of Patient-Reported and Performance-Based Measures of Function in the Boston RISE Study. J Gerontol A Biol Sci Med Sci. 2015 May 1;70(5):616–22.
5. Clemson L, Fiatarone Singh MA, Bundy A, Cumming RG, Manollaras K, O'Loughlin P, Black D. Integration of balance and strength training into daily life activity to reduce rate of falls in older people (the LiFE study): randomised parallel trial. BMJ. 2012;345:e4547.
6. Klenk J, Dallmeier D, Denkinger MD, Rapp K, Koenig W, Rothenbacher D. Objectively Measured Walking Duration and Sedentary Behaviour and Four-Year Mortality in Older People. PLoS One. 2016;11(4):e0153779.
7. Christofoletti G, McNeely ME, Campbell MC, Duncan RP, Earhart GM. Investigation of factors impacting mobility and gait in Parkinson disease. Hum Mov Sci 2016 Oct;49:308-14.
8. Lowry K, Woods T, Malone A, Krajek A, Smiley A & Van Swearingen J (2020): The Figure-of-8 Walk Test used to detect the loss of motor skill in walking among persons with Parkinson's disease, Physiotherapy Theory and Practice, DOI: 10.1080/09593985.2020.1774948
9. Strouwen C, Molenaar EALM, Münks L, Keus SHJ, Zijlmans JCM, Vandenberghe W, BloemBR, Nieuwboer A. Training dual tasks together or apart in Parkinson's disease:

Results fromthe DUALITY trial. Mov Disord. 2017 Aug;32(8):1201-1210. doi: 10.1002/mds.27014. Epub 2017 Apr 25. PMID: 28440888.
10. Storm FA, Nair KPS, Clarke AJ, Van der Meulen JM, Mazzà C (2018) Free-living and laboratory gait characteristics in patients with multiple sclerosis. PLoS ONE 13(5): e0196463. https://doi.org/10.1371/journal.pone.0196463
11. Nilsagård Y, Lundholm C, Denison E, Gunnarsson L-G. Predicting accidental falls in people with multiple sclerosis — a longitudinal study. *Clinical Rehabilitation*. 2009;23(3):259-269. doi:10.1177/0269215508095087
12. Mazumder R, Murchison C, Bourdette D, Cameron M (2014) Falls in People with Multiple Sclerosis Compared with Falls in Healthy Controls. PLOS ONE 9(9): e107620.
13. Demeyer H, Costilla-Frias M, Louvaris Z, Gimeno-Santos E, Tabberer M, Rabinovich R, de Jong C, Polkey M, Hopkinson N, Karlsson N, Serra I, Vogiatzis I, Troosters T, Garcia-Aymerich J. Both moderate and severe exacerbations accelerate physical activity decline in COPD patients. European Respiratory Journal Jan 2018, 51 (1) 1702110; DOI: 10.1183/13993003.02110-201
14. Arbillaga-Etxarri A, Gimeno-Santos E, Barberan-Garcia A, et al. Long-term efficacy and effectiveness of a behavioural and community-based exercise intervention (Urban Training) to increase physical activity in patients with COPD: a randomised controlled trial. Eur Respir J 2018; 52: 1800063 [https://doi.org/10.1183/13993003.00063-2018].
15. Donaire-Gonzalez D, Gimeno-Santos E, Balcells E, de Batlle J, Ramon MA, Rodriguez E, Farrero E, Benet M, Guerra S, Sauleda J, Ferrer A, Ferrer J, Barberà JA, Rodriguez-Roisin R, Gea J, Agustí A, Antó JM, Garcia-Aymerich J, the PAC-COPD Study Group. Benefits of physical activity on COPD hospitalisation depend on intensity. European Respiratory Journal Nov 2015, 46 (5) 1281-1289; DOI: 10.1183/13993003.01699-2014
16. Kon SSC, Jones SE, Schofield SJ, et alGait speed and readmission following hospitalisation for acute exacerbations of COPD: a prospective studyThorax 2015;70:1131-1137.
17. Svenøy S, Watne LO, Hestnes I, Westberg M, Madsen JE, Frihagen F. Results after introduction of a hip fracture care pathway: comparison with usual care. Acta Orthopaedica. 2020 Mar 3;91(2):139–45.
18. Guralnik JM, Simonsick EM, Ferrucci L, Glynn RJ, Berkman LF, Blazer DG, Scherr PA, Wallace RB. A short physical performance battery assessing lower extremity function: association with self-reported disability and prediction of mortality and nursing home admission. J Gerontol. 1994 Mar;49(2):M85-94.
19. Thingstad P, Taraldsen K, Saltvedt I, Sletvold O, Vereijken B, Lamb SE, Helbostad JL. The long-term effect of comprehensive geriatric care on gait after hip fracture: the Trondheim Hip Fracture Trial—a randomised controlled trial. Osteoporos Int. 2016 Mar 1;27(3):933–42.
20. The European Commission D-GfRI. H2020 Programme Guidelines on FAIR Data Management in Horizon 2020. 2016. Available from: https://ec.europa.eu/research/participants/data/ref/h2020/grants_manual/hi/oa_pilot/h2020-hi-oa-data-mgt_en.pdf.
21. Guidance for Industry, Electronic Source Data in Clinical Investigations. U.S. Department of Health and Human Services Food and Drug Administration, Center for Drug Evaluation and Research (CDER), Center for Biologics Evaluation and Research (CBER), Center for Devices and Radiological Health (CDRH); 2013 [cited 15 February 2017]. Available from: http://www.fda.gov/downloads/Drugs/GuidanceComplianceRegulatoryInformation/Guidances/UCM328691.pdf

22. Regulation (EU) 2016/679 of the European Parliament and of the Council on the protection of natural persons with regard to the processing of personal data and on the free movement of such data, and repealing Directive 95/46/EC (General Data Protection Regulation); 2016 [cited 15 February 2017]. Available from: http://eur-lex.europa.eu/legal-content/EN/TXT/PDF/?uri=CELEX:32016R0679&from=EN.
23. Directive 2006/24/EC of the European Parliament and of the Council on the retention of data generated or processed in connection with the provision of publicly available electronic communications services or of public communications networks and amending Directive 2002/58/EC; 2006 [cited 15 February 2017]. Available from: http://eur-lex.europa.eu/legal-content/EN/TXT/PDF/?uri=CELEX:32006L0024&from=en.
24. The European Code of Conduct for Research Integrity 2017. Available from: https://ec.europa.eu/research/participants/data/ref/h2020/other/hi/h2020-ethics_code-of-conduct_en.pdf

## 15. APPENDICIES

### Appendix 1: Schedule of Procedures

| Outcome Measures | BL (T1) | FU (T2) | FU (T3) | FU (T4) | FU (T5) |
|---|---|---|---|---|---|
| Time point | M0$ | M6 (±2 weeks) | M12 (±4 weeks) | M18 (±4 weeks) | M24 (±4 weeks) |
| Location | Lab | Lab | Lab | Lab | Lab |
| Year of birth | X | | | | |
| Gender | X | | | | |
| Height | X | | | | X |
| Weight | X | | X | | X |
| Shoe size (EU) | X | | | | |
| Leg length | X | | | | |
| Education | X | | | | |
| Employment | X | | X | | X |
| Marital status | X | | X | | X |
| Living arrangements (Place of residence) | X | X | X | X | X |
| Overall health status (single item) | X | | X | | X |
| Smoking history | X | | | | |

| | | | | | |
|---|---|---|---|---|---|
| Alcohol consumption | X | | X | | X |
| Ethnicity | X | | | | |
| Falls history at baseline (12M) | X | | | | |
| Fracture history at baseline (12M) | X | | | | |
| Sensory impairment - Vision (Snellen Chart) | X | | | | |
| COVID-19 Questionnaire | X | X | X | X | X |
| Activities and Participation –<br>Later Life Functional Disability Index (LLFDI) | X | X | X | X | X |
| Mortality | | X | X | X | X |
| Nursing home admission | | X | X | X | X |
| Hospital admission | | X | X | X | X |
| Falls and injuries | | X | X | X | X |
| Medication (at baseline and changes) | X | X | X | X | X |
| Non-pharmacological interventions (at baseline and changes) | X | X | X | X | X |
| Blood pressure – general question | X | | X | | X |
| Health related quality of life - EQ-5D-5L | X | | X | | X |
| Pain - Visual Analogue Scale (VAS)<br>during rest and walking | X | X | X | X | X |
| Comorbidities –<br>Groll Functional Comorbidity Index (FCI) | X | | | | X |
| Frailty Index (FI) by Fried | X | | | | X |
| Fatigue (FACIT) | X | | X | | X |
| Minimal important difference (MID) (anchor question) | | X | X | X | X |
| Bioimpedance (BIA) | X | | | | X |
| Use of mobility aids<br>Indoor and outdoor | X | X | X | X | X |
| Short Physical Performance Battery (SPPB) | X | X | X | X | X |
| Muscular Strength - Hand Grip | X | | X | | X |
| Timed Up-and-Go (TUG) | | X | X | X | X |

| | | | | | |
|---|---|---|---|---|---|
| 6 Minute Walking Test in 20 m corridor (6MWT) | X | X | X | X | X |
| Digital Mobility Assessment<br>(7day measurement) | X | X | X | X | X |
| Univ. of Alabama at Birmingham Life Space Ass. (LSA) **for community dwellers** | | X | X | X | X |
| Nursing home life space diameter<br>**Only nursing home residents** | | X | X | X | X |
| Falls Efficacy Scale International Short Form<br>(Short FES-I) | X | | X | | X |
| Depression – Patient Health Questionnaire (PHQ-2) | X | | X | | X |
| Social isolation and loneliness | X | | X | | X |
| Cognitive function – Mini-Mental State Examination Short version (SMMSE) | X | | | | X |

| | | | | | |
|---|---|---|---|---|---|
| **Disease specific measures** | | | | | |
| ***Parkinson's disease measures*** | | | | | |
| Unified Parkinson's Disease Rating Scale (MDS-UPDRS) I-IV | X | | X | | X |
| Movement Disorders Society Unified Parkinson's Disease Rating Scale (MDS-UPDRS) part III (includes Hoehn & Yahr) | | X | | X | |
| Mini balance Evaluation Systems Test (Mini-BEST) | X | | X | | X |
| New Freezing of Gait Questionnaire (NFOGQ) | X | X | X | X | X |
| Montreal Cognitive Assessment (MoCA) | X | | X | | X |
| ***Multiple Sclerosis*** | | | | | |
| Modified Impact Fatigue Scale (MIFS) | X | X | X | X | X |
| Multiple Sclerosis Functional Composite (MSFC) | X | X | X | X | X |
| Expanded Disability Status Scale (EDSS) | X | X | X | X | X |

| | | | | | |
|---|---|---|---|---|---|
| Patient determined disease steps (PDDS) scale | X | X | X | X | X |
| Multiple sclerosis walking scale-12 (MSWS-12) | X | X | X | X | X |
| Symbol Digit Modalities Test (SDMT) | X | X | X | X | X |
| Low-contrast letter acuity | X | X | X | X | X |
| ***Chronic obstructive pulmonary disease*** | | | | | |
| Lung function (spirometry) (FEV-1, FVC and FEV1/FVC ratio) | X | X | X | X | X |
| Exacerbations<br>in previous 12 months | X | | | | |
| Exacerbation<br>in previous 6 months | | X | X | X | X |
| 2nd Six minute walking test (6MWT) | X | X | X | X | X |
| Change in smoking / use of e-cigarettes | | X | X | X | X |
| COPD assessment test (CAT) | X | | X | | X |
| Modified Medical Research Council (mMRC) dyspnoea scale | X | X | X | X | X |
| PROactive Physical Activity in COPD clinical visit version (C-PPAC) | X | X | X | X | X |
| Isometric quadriceps muscle force | X | | X | | X |
| Oxygen saturation at rest | X | | X | | X |
| ***Proximal femoral fracture*** | | | | | |

| | | | | | |
|---|---|---|---|---|---|
| Descriptive (Fracture type, operation type, treatment) | X | | | | |
| American Society of Anaesthesiologists Classification (ASA) score | X | | | | |
| Prefracture Barthel Index (BI) | X | | | | |
| Barthel Index (BI) | | | X | | X |
| Prefracture Nottingham Extended ADL Index (NEADL) | X | | | | |
| Nottingham Extended ADL Index (NEADL) | | | X | | X |
| 4AT Delirium Scale | X | | | | |
| Pre-fracture Clinical Dementia Rating scale (CDR) | X | | | | |
| Clinical Dementia Rating scale (CDR) | | | X | | X |
| Montreal Cognitive Assessment (MoCA) | | X | | | X |
| Subjective Hearing | X | | | | X |
